# PSD95 and nNOS interaction as a novel molecular target to modulate conditioned fear: relevance to PTSD

**DOI:** 10.1038/s41398-018-0208-5

**Published:** 2018-08-14

**Authors:** L.- P. Li, E. T. Dustrude, M. M. Haulcomb, A. R. Abreu, S. D. Fitz, P. L. Johnson, G. A. Thakur, A. I. Molosh, Y. Lai, A. Shekhar

**Affiliations:** 1 Medical Neuroscience Graduate Program, Indiana University School of Medicine, Indianapolis, IN USA; 2 Stark Neurosciences Research Institute, Indiana University School of Medicine, Indianapolis, IN USA; 3 Department of Psychiatry, Indiana University School of Medicine, Indianapolis, IN USA; 4 Anagin Inc., Indiana Center for Biomedical Innovation, Indianapolis, IN USA; 5 Department of Anatomy and Cell Biology, Indiana University School of Medicine, Indianapolis, IN USA; 6 Department of Pharmaceutical Sciences, Northeastern University, Boston, MA USA; 7 Gill Center for Biomolecular Science and Department of Psychological and Brain Sciences, Indiana University Bloomington, Bloomington, IN USA; 8 Indiana Clinical and Translational Sciences Institute, Indiana University School of Medicine, Indianapolis, IN USA

## Abstract

Stimulation of N-methyl-D-aspartic acid receptors (NMDARs) and the resulting increase of nitric oxide (NO) production are critical for fear memory formation. Following NMDAR activation, efficient production of NO requires linking the 95 kDa postsynaptic density protein (PSD95), a scaffolding protein to neuronal nitric oxide synthase (nNOS). A variety of previously studied NMDAR antagonists and NOS inhibitors can disrupt fear conditioning, but they also affect many other CNS functions such as motor activity, anxiety, and learning. We hypothesized that disrupting nNOS and PSD95 interaction in the amygdala, a critical site for fear memory formation, will reduce conditioned fear. Our results show that systemic treatment with ZL006, a compound that disrupts PSD95/nNOS binding, attenuates fear memory compared to its inactive isomer ZL007. Co-immunoprecipitation after fear conditioning showed a robust increase in the amygdala PSD95/nNOS binding, which was blocked by systemic pre-administration of ZL006. Treatment of amygdala slices with ZL006 also impaired long-term potentiation (LTP), a cellular signature of synaptic plasticity. Direct intra-amygdala infusion of ZL006 also attenuated conditioned fear. Finally, unlike NMDAR antagonist MK-801, ZL006 does not affect locomotion, social interaction, object recognition memory, and spatial memory. These findings support the hypothesis that disrupting the PSD95/nNOS interaction downstream of NMDARs selectively reduces fear memory, and highlights PSD95/nNOS interaction as a novel target for fear-related disorders, such as posttraumatic stress disorder.

## Introduction

Normal fear learning and memory allow animals to predict and avoid physical dangers and are therefore essential to survival. However, following traumatic experiences, these mechanisms can lead to symptoms of syndromes such as posttraumatic stress disorder (PTSD)^1,2^. PTSD is a severe psychiatric disorder in which fear responses are likely sustained, generalized, and inappropriately triggered out of context^1,2^. Pavlovian fear conditioning is a well-established laboratory model of fear learning that is often used to elucidate mechanism of fear acquisition and extinction. In this paradigm, a neutral event (a conditioned stimulus, (CS)), such as a tone, is paired with an aversive event (an unconditioned stimulus (US)), such as a footshock. Once learned, the CS acquires the ability to evoke fear responses, such as freezing in anticipation of the US^3^.

Pavlovian fear conditioning is known to be dependent on the synaptic plasticity within the amygdala^4,5^ and is mediated by excitatory neurotransmission acting through N-methyl-D-aspartic acid receptors (NMDARs). A number of studies have demonstrated a critical role of NMDARs in fear conditioning. For example, systemic and CNS site-specific administration of NMDAR antagonists block fear acquisition when given before training^6–9^ and impair fear expression when administered before fear recall^10,11^. Unfortunately, despite this important role for NMDARs in impairing fear formation, NMDAR antagonists have limited therapeutic potential due to their significant adverse side-effect profiles^12,13^.

Stimulation of NMDARs activates a number of downstream signaling pathways. One such downstream effect involves activation of the enzyme neuronal nitric oxide synthase (nNOS) and the resulting production of the signaling molecule nitric oxide (NO). nNOS is one of three isoforms of NOS (the other isoforms being endothelial NOS and inducible NOS) and is preferentially expressed in neurons and functionally coupled to NMDAR signaling^14^. There is strong evidence that activation of nNOS following NMDAR activation is a critical component of fear memory formation^15,16^. Indeed, pharmacological inhibition of enzyme activity and gene deletion of nNOS have been shown to reduce fear. For example, systemic and intra-amygdala administration of NOS inhibitors reduce fear memories in multiple models of fear conditioning^17,18^; mice with nNOS gene knockout display impairments in both contextual and cued fear learning^19^. Despite being downstream of NMDARs, unfortunately, global inhibition of nNOS enzyme itself cause undesired systemic effects, such as deficits in motor functions^20–22^ and impairments in some other forms of learning^23–26^. Therefore, similar to direct NMDAR antagonism, therapeutic targeting of the downstream nNOS enzyme is undesirable due to adverse effects.

Following NMDAR activation, nNOS binds to the scaffolding protein postsynaptic density protein 95 kDa (PSD95), and this is a required step for the efficient production of NO^14^. Thus, selective disruption of the PSD95/nNOS binding would allow a targeted approach to specific reduction of NO production during high glutamate neurotransmission state without affecting normal intracellular nNOS functions. By not disrupting NMDAR-dependent signaling pathways, this approach could circumvent the adverse effects associated with catalytic nNOS inhibitors or NMDAR antagonists.

In the present study, we hypothesized that disrupting PSD95/nNOS interaction would reduce fear, similar to the NMDAR antagonists but with a better adverse effect profile.

To explore this hypothesis, we first examined the effect of inhibition of PSD95/nNOS interaction on fear memory formation by utilizing a small molecule disruptor of PSD95/nNOS interaction ZL006 (4-(3,5-Dichloro-2-hydroxy-benzylamino)-2-hydroxybenzoic acid). ZL006 is a structural analog of the small molecule 2-((1H-benzo [d] [1,2,3] triazol-5-ylamino) methyl)-4,6-dichlorophenol (IC87201), the first reported disruptor of PSD95/nNOS interaction. IC870201 was first identified in a high throughput screen using the purified PDZ domains of PSD95 and nNOS in a protein–protein binding assay^27^. ZL006 was synthesized based on the molecular determinants required for PSD95 and nNOS interaction^28^ and has also been verified to selectively disrupt PSD95/nNOS interaction without affecting PSD95 interactions with other proteins^28,29^.

In a series of studies, we tested if systemic ZL006 given shortly after a conditioning session would impair fear memory consolidation in auditory fear conditioning model, without affecting locomotor function, anxiety, and other types of memory tests. Next, we studied the cellular and molecular mechanisms mediating the effects of ZL006 on fear memory formation. By using co-immunoprecipitation (CO-IP) techniques, we tested if fear conditioning results in significant increases in PSD95/nNOS binding within the amygdala in a time dependent manner, and if ZL006 disrupts this binding. Utilizing brain slice electrophysiology, we tested whether ZL006 disrupts LTP in the amygdala. Finally, to determine if disruption of PSD95/nNOS binding within the amygdala is sufficient to attenuate fear we directly administered ZL006 within the amygdala to test if it would attenuate conditioned fear. Our findings reported here support the hypothesis that PSD95/nNOS interaction in the amygdala is a key step in fear memory formation and may represent a novel treatment target with a minimal adverse effect profile for fear-related disorders, such as PTSD.

## Materials and methods

### Animals

Behavioral and biochemical experiments were performed in adult male Sprague–Dawley rats (250–300 g, Harlan, IN). The rats were housed singly in a temperature-controlled room (22 °C) on a 12-hour light/dark cycle (lights on at 0700 h) and left to acclimate to housing for at least 3 days following delivery. Food and water were provided ad libitum. The rats were handled daily for a minimum of 3 days before any behavioral experiment. Animal care procedures were conducted in accordance with the NIH Guidelines for the Care and Use of Laboratory Animals, 8th Edition and approved by the IUPUI Institutional Animal Care and Use Committee.

### Behavioral tests

#### Cued fear conditioning and memory expression testing

The day prior to conditioning, the rats were handled and habituated to the conditioning box (25.5 × 25.5 × 39.5 cm) for 10 minutes. The conditioning box was situated in a larger sound-attenuated chamber, which was illuminated with a white 15-Lux light. A speaker in the rear wall of the chamber was operating during all sessions to provide white noise. The floor of the conditioning box was constructed of parallel stainless-steel bars and connected to a scrambled shock generator (Stoelting Co., Wood Dale, IL, USA). Before each trial, the chamber and the conditioning box were cleaned with 70% ethanol to remove olfactory cues. On the conditioning day, the rats were trained with three conditioning trials. Each trial consisted of a 20 s, 4 kHz, 80 dB tone that co-terminated with a 0.5 s, 0.8 mA footshock (inter-trial interval (ITI) 120 s). Rats were allowed to explore the chamber for 100 s before conditioning began and remained in the chamber for 60 s after the last trial. “Tone only” control rats were placed in the conditioning box and exposed to 3 tones (20 s, 4 kHz, 80 dB) without receiving shocks. Immediately after fear conditioning, the animals received treatments of drugs or vehicle. Testing for conditioned fear responses (freezing) in rats were conducted 24 h following conditioning. For this test, the rats were exposed to ten CS tones (4 kHz, 80 dB, 20 s, ITI 60 s). Total time freezing during the CS presentations were recorded and scored manually by blinded raters, and this number was expressed as a percentage of the total CS. Freezing was defined as the absence of all movement except for normal breathing.

#### Novel object recognition test

Novel object recognition test (NORT) was performed according to the method described previously^30^, with some minor modifications. The experiments were carried out in an open-field box measuring 100 × 100 × 20 cm. Prior to testing, the rats were allowed to explore the box for 5 min per day for 3 consecutive days with no objects present. Testing consisted of two 2 min trials. During the familiarization trial, a rat was placed in the box containing two identical objects (plastic cylinders 6 cm in diameter and 12 cm tall in white and red) in two opposite corners and released against the center of the opposite wall with its back to the objects. This was done to prevent coercion to explore the objects^31^. The animals were regarded to be exploring when they were facing, sniffing, or biting the object with nose and/or forepaws. Immediately after familiarization, the rats received intraperitoneal (i.p.) injections of vehicle or drugs and were returned to its home cage. After a waiting period of 3 h (ITI = 3 h), the rat was placed in the box again and test trial was performed. During this trial, a new object (plastic building block in yellow or green, 7 × 3.5 × 9 cm) replaced one of the familiar objects used in the familiarization trial. The times spent in exploring each object during both trials were recorded manually by using a stopwatch. The box and the objects were cleaned with 70% of ethanol between trials. Discrimination index (DI) used to measure the discrimination behavior was calculated as the difference in exploration time for the novel (T_N_) versus familiar objects (T_F_), then dividing this value by the total time spent exploring the two objects in the test trial. *D*_*I*_ = *T*_*N*_ − *T*_*F*_ / *T*_*N*_+*T*_*F*_^32^.

#### Y-maze

Y-maze task was performed as previously described^33^. Y-maze was constructed of Plexiglass with 3 arms each measuring 34 × 8 × 14.5 cm. Visual cues were placed on the walls of the maze. The maze was located in a room with a light of 350 Lux brightness. Numerous distal cues (tables, computers, chairs, and various small objects) were around the Y-maze in the room and were kept constant during the entire behavioral testing period. The three arms were randomly designated: start arm, in which the rats started to explore (always open), novel arm, which was blocked during the 1st trial, but open during the 2nd trial, and other arm (always open). The floors and walls of the maze were cleaned with 70% ethanol to remove olfactory cues. The Y-maze test consisted of two trials: acquisition trial and test trial. In the acquisition trial, the rat was allowed to explore only two arms (the start arm and the other arm) of the maze for 10 minutes. Injections (i.p.) of vehicle or drugs were given just after acquisition trial. After 1 h waiting period (ITI = 1 h), the test trial was performed. In this trial, the novel arm was opened and the rats were allowed 5 minutes to explore all three arms. By using a ceiling-mounted CCD camera, all trials were recorded. Video recordings were later analyzed and the number of entries and time spent in each arm were scored manually for each rat.

#### Open-field test

The open-field (OF) apparatus consisted of a plexiglass open-topped chamber (91.5 × 91.5 × 30.5 cm), a ceiling-mounted CCD camera and a 25 W red light bulb were placed 2 meters above the center of the chamber. One hour after vehicle or drug treatment, rats were gently placed in the center and allowed to freely move 5 minutes while being tracked by an automated tracking system (ANY-MAZE, Stoelting Co., Wood Dale, IL, USA). Total distance traveled was used to measure locomotion activity and results were normalized to vehicle controls.

#### Social interaction test

Social interaction (SI) test was performed 5 min after OF test in the same apparatus. The protocol used for the SI test has been described previously^34,35^. In brief, the “experimental” rat and the “partner” rat were simultaneously placed into the chamber for a 5 min test. The “partner” rat was age-, sex- and weight-matched to the “experimental” rat. All tests were video recorded from above and then manually scored using using ODlog for Mac OS X version 2.6.1. Social interaction time (in s) per pair of rats was measured as time spent by the “experimental” rat engaging in non-aggressive physical investigation of the “partner” rat; defined by the “experimental” rat sniffing, climbing over and crawling under the “partner” rat, mutual grooming, genital investigation, or following and walking around the partner.

#### Randomization and blinding

All behavioral tests performed on each animal were determined randomly by an experimenter. No explicit randomization algorithm was used. In the behavioral tests where manual scoring was required, the tests were video recorded and scored by an experimenter who was blinded to the treatments.

### Co-immunoprecipitation

Following behavioral training and sacrifice by decapitation under isoflurane, the brains were removed and immediately frozen in iso-pentane (Fisher Scientific, Rockford, IL, USA) on dry ice and were stored at −80 °C until processed. Punches containing the BLA were obtained using a 1 mm diameter Harris micro-punch (Electron Microscopy Sciences, Hatfield, PA, USA) from 300-μm thick sections taken on a freezing microtome (see Supplementary Fig. S1 for locations of micropunches). The punches were immediately homogenized in 100 μl of ice-cold lysis buffer (25 mM Tris, 150 mM NaCl, 1 mM EDTA, 1% NP-40, 5% glycerol, PH 7.4) supplemented with Halt protease inhibitor cocktail and Halt phosphatase inhibitor cocktail (Thermo Scientific, Rockford, IL, USA). After lysis on ice for 15 min with periodic mixing, samples were centrifuged at 13,000× *g*/4 °C for 15 min. The supernatants were pre-incubated for 1 h at 4 °C with 25 μl of Control Agarose Resin (Thermo Scientific) and then centrifuged to remove proteins that adhered nonspecifically to the resin. nNOS antibody (mouse antibody to nNOS, A-11, Santa Cruz Biotechnology, Dallas, TX, USA) at 2 μg per 100 μg of total protein or normal mouse IgG (Santa Cruz Biotechnology) was added to the supernatant and incubated overnight at 4 °C. Protein A/G Agarose (Thermo Scientific) was then added to the antibody/lysate sample and incubated at 4 °C for 1 h. Immune complexes were isolated by centrifugation, washed 5 times with lysis buffer, and bound proteins were eluted by heating at 95 °C in loading buffer for 10 min for immunoblotting. Samples were loaded to 10% acrylamide denaturing gels (SDS-PAGE) and transferred to nitrocellulose membranes (Amersham, Pittsburgh, PA, USA). Nitrocellulose membranes were blocked with 5% milk in TBST buffer (50 mM Tris-Cl, pH 7.6; 150 mM NaCl; 0.1% Tween 20) and then incubated with mouse nNOS antibody (1:1000, Santa Cruz Biotechnology), mouse PSD95 antibody (1:2000, Invitrogen, Rockford, IL, USA), and mouse β-actin antibody (1:10000, Santa Cruz Biotechnology) and detected using goat anti-mouse-HRP (Santa Cruz Biotechnology) at 1: 2,000 for nNOS, 1: 5000 for PSD95, 1: 10000 for β-actin. Detection of protein band signals was achieved by adding chemoluminescent buffer (Millipore, Billerica, MA, USA) to the blots. Films were scanned and densitometry was performed using ImageJ 1.48 software.

### Surgery

Prior to surgery, the rats were anesthetized by placing them in a closed plastic box connected to an Isoflurane system (MGX Research Machine, Vetamac, Rossville, IN, USA). The animals were then removed from the box and placed on a stereotaxic instrument (Kopf Instruments, Tujunga, CA, USA). Anesthesia was maintained via a nose cone, which allowed for constant flow of isoflurane (2–3% by volume) throughout the surgery. An incision was made in the scalp and the skull was cleaned and dried. Two stainless-steel guide cannulas (26 gauge, Plastics One, Roanoke, VA, USA) were implanted bilaterally into the basolateral nucleus of the amygdala (BLA) using the following coordinates, anterior, −2.3 mm; lateral, ±4.9 mm; and ventral, −7.4 mm, according to the brain atlas of Paxinos and Watson (fifth edition). The guide cannulas were secured into place using three 2.4 mm screws anchored into the skull along with cranioplastic cement. Dummy cannulas (Plastics One, Roanoke, VA, USA) with lengths matching the guide cannulas were placed inside the guide cannulas to prevent occlusions. Following surgery, all rats were given pain medication (buprenorphine, Indiana University School of Medicine Laboratory Animal Resources) and allowed to recover for 7 days before behavioral testing. During recovery, the rats were gently handled every day for a minimum of 2 min.

### Intracranial injections

To execute local infusions into BLA, the dummy cannulas were quickly removed from the guide cannulas and were replaced by internal cannulas (extended 1.0 mm beyond the guide cannulas, Plastics One, Roanoke, VA, USA). The internal cannulas were connected via polyethylene tubing to 10 µl microsyringes (Hamilton, Reno, NV, USA). An injection volume of 0.25 µl was delivered using a Harvard PHD 2000 (Harvard Apparatus, Inc., South Natick, MA, USA) syringe pump over the course of 2.5 min. Internal cannulas remained in the guide cannulas for 1 min after druginfusion to allow diffusion of the drug from the tip. At the end of the microinfusion experiment, the rats were enuthanized by an overdose of isoflurane and perfused with 4% Paraformaldehyde (PFA). Neutral red staining and light microscopy were used to verify the location of the cannula tips within the BLA (see Supplementary Fig. S3 for details).

### Slice electrophysiology

Electrophysiological experiments in amygdala slices were conducted as previously described^36^. The rats were anesthetized with 3–5% isoflurane and decapitated. The brains were rapidly removed and placed in ice-cold oxygenated artificial cerebrospinal fluid (ACSF) containing in mM: 130 NaCl, 3.5 KCl, 1.1 KH_2_PO_4_, 1.3 MgCl_2_, 2.5 CaCl_2_, 30 NaHCO_3_, 10 glucose (315 mOsm, 7.4 pH), and coronal slices (350 µM) were prepared containing the BLA (BLA, ~−2.3 mm from bregma). Slices were then subjected to a recovery protocol to improve cell viability which included thirty minute incubations in 30 °C ACSF and then room temperature ACSF prior to recording. Oxygenated ACSF, heated to 30 °C, was perfused at a rate of 2–3 ml per minute during recording on the stage of a Nikon E600FN Eclipse microscope (Nikon Instruments, Melville, NY, USA). Borosilicate glass electrodes (WPI, Sarasota, FL, USA) with resistances between 3 and 6 MΩ were filled with potassium gluconate based recording solution containing in mM: 130 K-Gluconate, 3 KCl, 3 MgCl2, 5 phosphocreatine, 2 K-ATP, 0.2 Na-GTP, 10 HEPES, 0.05 picrotoxin (Sigma, St. Louis, MO, USA), and whole-cell patch clamp responses in current clamp mode were recorded by standard techniques using Multiclamp700B amplifier and Digidata1440 digitizer (Molecular Devices, Sunnyvale, CA, USA). BLA pyramidal neurons were identified by their morphology and further validated by basic electrophysiological property of input resistance (~35 MΩ)^37^. Cell-holding potential was maintained at −70 mV and electrically evoked responses were produced by Master8 pulse stimulator (A.M.P.I, Jerusalem, Israel) as previously described^38^. Compounds were added directly to the ACSF and included ZL-family compounds at 10 µM and GABA-B receptor antagonist CGP52432 (Tocris, Minneapolis, MN, USA) at 1 µM. Evoked EPSPs were generated via electrical stimulation with a concentric, platinum/iridium, bipolar electrode (FHC, Bowdoin, ME, USA) placed ~1 mm from the recorded cell, within the BLA and directly medial to the external capsule. Baseline evoked EPSP responses were recorded once per minute for 10 minutes at the beginning of each experiment to verify consistent cell properties of resistance and evoked response amplitude. For conditions where ZL-family compounds were tested, an additional 10 minutes of baseline responses were recorded to determine if the compound had any effect on evoked response amplitude. Current injected for evoked responses was adjusted for each individual cell to produce roughly 5 mV depolarization and reliably allowed detection of potentiation without depolarization to action potential threshold. The average current injected to achieve this depolarization was 290 pA. For cells that demonstrate a consistent baseline, 100 Hz high frequency stimulation (HFS) was applied to induce potentiation of evoked responses as previously described^39^. This combination of depolarization and evoked stimulation produces action potentials and when preformed in 20 one-second bursts spaced 20 seconds apart is able to produce short-term and LTP. Cells that did not induce short-term potentiation were omitted.

### Drugs and chemicals

ZL006 and MK-801 were purchased from Sigma Aldrich (St. Louis, MO, USA). ZL007 was synthesized in the laboratory of Dr. Ganesh Thakur at the Northeastern University Center for Drug Discovery (Boston, MA, USA). In the behavioral tests with i.p. injections, ZL006 and ZL007 were dissolved in a vehicle of 10% DMSO (Sigma Aldrich, St. Louis, MO, USA), with the remaining 90% consisting of 100% ethanol (Fisher Scientific, Rockford, IL, USA), emulphor (Alkamuls EL-620, Solvay, Brussels, Belgium) and sterilized saline at a ratio of 1:1:8, respectively. MK-801 was dissolved in sterilized saline. Injection volume was 1 ml/kg and control animals were injected with an equal volume of vehicle. For intra-amygdala infusion, ZL006 was diluted from ZL006 stock solution (dissolved in 100% DMSO) at 1:1000 in ACSF to yield a final concentration of 10 µM.

### Statistical analysis

Data were expressed as group mean with SEM. Sample size was determined based on power calculations from previous reports and/or our pilot studies. A two-way repeated ANOVA was used to compare the effects of treatment and time across the trials of fear conditioning and memory expression tests, followed by post hoc Fisher’s LSD tests. For multiple group comparisons, statistical differences were calculated by one-way ANOVA followed by post hoc Fisher’s LSD tests. Greenhouse–Geisser correction was applied when the assumption of sphericity was violated. Data from slice electrophysiology was analyzed by two-way ANOVA comparing differences between treatments and over time. Statistical significance was determined using Fisher’s LSD multiple comparisons post hoc analysis. Values of *P* < 0.05 were considered significant.

### Experimental procedures

#### Effects of systemic disruption of PSD95/nNOS interactions on consolidation of auditory Pavlovian fear conditioning

**Fig. 1:**
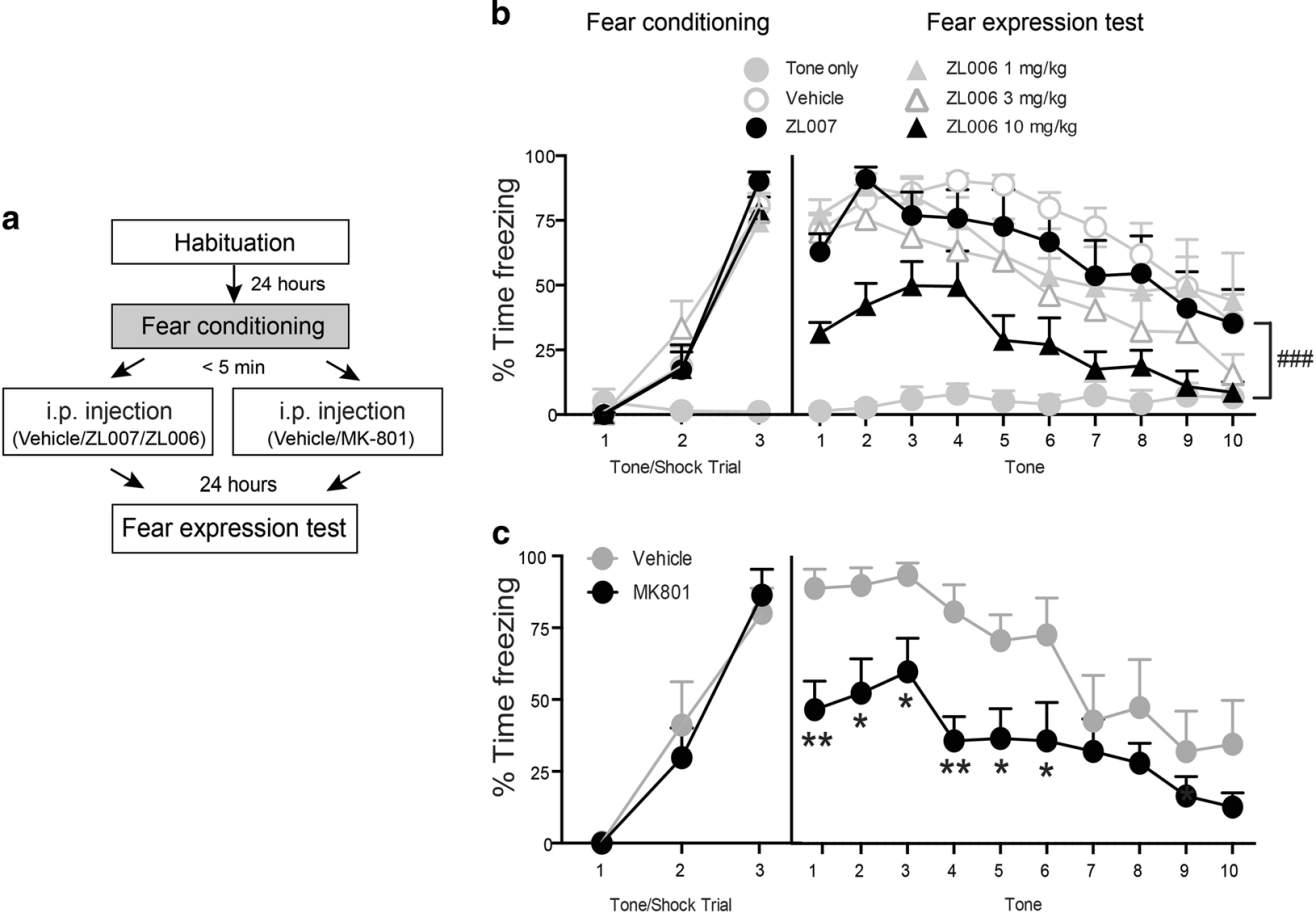
Systemic disruption of PSD95/nNOS interaction and inhibition of NMDAR impaired consolidation of auditory Pavlovian fear conditioning. **a** Schematic of the behavioral protocol. Immediately after fear conditioning, rats were given i.p. injections of indicated treatments. The retention of conditioned fear memory was tested 24 h later. **b** The five groups of animals that had tone/shock pairings showed normal cued fear acquisition (Trial: *F*_2, 56_ = 299.4, *P* < 0.0001). In the fear expression test, 10 mg/kg ZL006 treated animals (*n* = 8) displayed significantly decreased freezing responses when compared with vehicle controls (*n* = 7) (post hoc test: *t* = 3.77, DF = 28, *P* < 0.001). ### *P* < 0.001 ZL006 10 mg/kg vs. vehicle; Groups with lower doses of ZL006 (1 mg/kg and 3 mg/kg, *n* = 6) and ZL007 group (*n* = 6) showed similar levels of freezing during fear expression compared to the vehicle group (*P* > 0.05). Rats in “Tone only” group (*n* = 7) did not freeze to the CS in neither fear training nor fear expression test. **c** Both vehicle (*n* = 7) and MK-801 (*n* = 7) treated animals showed normal cued fear acquisition (Trial: *F*_2, 24_ = 59.62, *P* < 0.0001). However, MK-801 treated animals showed significantly decreased freezing responses in fear expression test (Treatment: *F*_1, 12_ = 8.550, *P* < 0.05). *P < 0.05, **P < 0.01 relative to vehicle group

To investigate if disruption of PSD95/nNOS binding can impair conditioned fear, we treated rats with i.p. injections of different doses of a small molecule inhibitor of PSD95/nNOS interaction, ZL006 (1 mg/kg, 3 mg/kg, or 10 mg/kg) immediately after training and tested fear expression 24 h later (Fig. 1a). In these tests, we utilized a control group of animals that received the same conditioning procedures but without shock pairing (“Tone only”). During the conditioning, shocked animals who acquire fear would freeze during the presentation of tone whereas “Tone only” controls would not freeze to the tone.

#### Effects of disrupting PSD95/nNOS on motor activity and short-term memory

While NMDA antagonists like MK-801 and ketamine are also highly effective in blocking fear conditioning, they have significant acute motor and memory effects. Therefore, we tested motor activity and short-term memory effects of disrupting PSD95-nNOS interactions using the dose of ZL006 that were effective in reducing fear memory (10 mg/kg), comparing it to similarly effective doses of MK-801 (0.1 mg/kg).

#### Determining fear-conditioning-induced increases in PSD95/nNOS binding in the amygdala, and the effects of pre-administration of ZL006

We sought to characterize the molecular mechanisms that underlie ZL006 action in attenuating memory formation of conditioned fear. Amygdala, and specifically, the BLA, is a critical neural locus for conditioned fear learning and expression^2,40,41^. We hypothesized that if PSD95/nNOS interaction is a critical downstream event from NMDAR signaling during fear conditioning, then an increased binding of these two proteins will be seen in the BLA following fear acquisition, and that this binding can be prevented by pre-administration of ZL006. To test this hypothesis, animals that received fear conditioning were sacrificed and the levels of PSD95/nNOS binding in the BLA were quantified with CO-IP with nNOS antibody followed by immunoblotting with nNOS and PSD95 antibodies. This CO-IP experiments after fear conditioning was repeated for several time points following fear conditioning ranging from 0.5 to 6 h. As a control, a separate group of rats received the same procedure but without shock pairing were sacrificed 0.5 h after no shock training (“Tone only”) (Fig. 3a).

#### Testing the effects of disrupting PSD95/nNOS binding on LTP of BLA neurons

Alterations of synaptic plasticity in the BLA have been thought to promote the induction and expression of fear memory^5,42^. To further investigate the mechanisms of ZL006 action in attenuating memory formation of conditioned fear, we examined whether local application of PSD95/nNOS binding inhibitor ZL006 would alter synaptic plasticity in the BLA slice preparations. We used whole-cell patch clamp technique and HFS protocol to induce LTP of BLA projection neurons and 10 µM ZL006 treated neurons were compared to neurons treated with vehicle or inactive isomer 10 µM ZL007.

#### Effects of disrupting PSD95/nNOS binding directly in the amygdala on fear conditioning

Finally, we asked if direct injections of PSD95/nNOS binding inhibitor ZL006 into BLA could impair memory formation of conditioned fear. The rats were implanted with guide cannulas into the BLA and underwent fear conditioning training seven days after surgery. Intra-BLA infusions of vehicle or 10 µM ZL006 were given immediately after training and rats were tested for expression of fear memory 24 h after training (Fig. 5a).

## Results

### Disruption of PSD95/nNOS interactions with ZL006 impairs fear conditioning

When given systemically immediately following fear conditioning sessions, we found that vehicle controls showed robust conditioned fear responses during the testing sessions 24 h later, whereas 10 mg/kg ZL006 treated group had significantly reduced conditioned freezing (multiple comparisons post hoc test, *P* < 0.05) (Fig. 1a, b). Lower doses of ZL006 failed to reduce freezing responses in this test. ZL007, an inactive analog of ZL006 was utilized as a negative control in these tests. We found that animals treated with 10 mg/kg ZL007 did not show differences in freezing responses when compared with vehicle controls (Fig. 1b). Similar to ZL006 (10 mg/kg), post-training i.p. administration of NMDAR antagonist MK-801 (0.1 mg/kg) also significantly reduced freezing responses in the fear expression test (Fig. 1a, c). Collectively, these findings suggest that similar to NMDAR antagonist MK-801, disruption of PSD95/nNOS binding can impair memory formation of auditory Pavlovian fear conditioning.

### ZL006 does not cause behavioral and memory disruptions seen with NMDAR antagonism

**Fig. 2:**
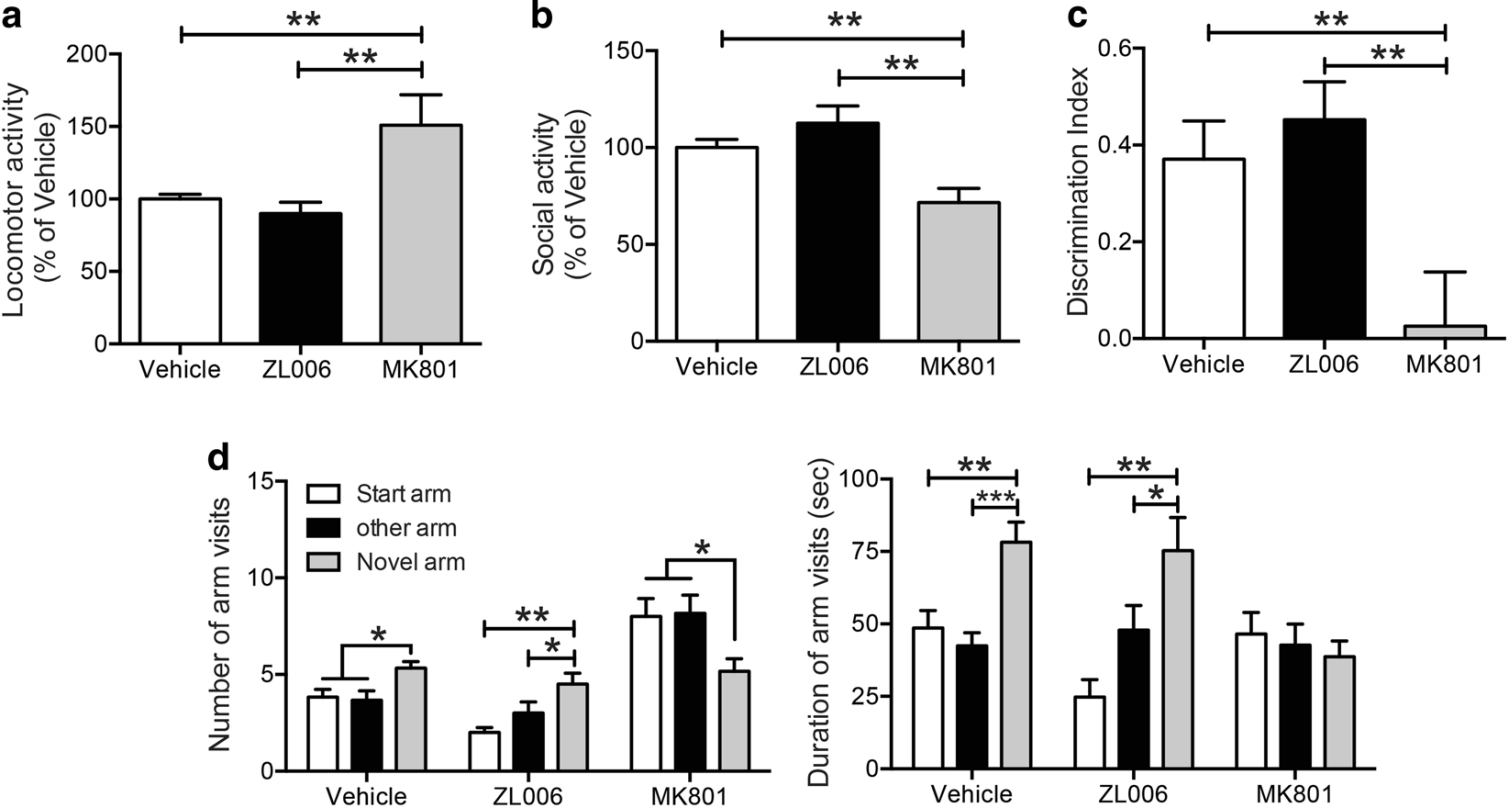
Disruption of PSD95/nNOS with ZL006 does not have the non-specific behavioral effects seen with NMDARs antagonist MK-801. **a** Animals received i.p. injections 1 h prior to the OF Test. MK-801-treated animals displayed increased locomotor activity (*F*_2, 25_ = 7.562, *P* < 0.01). SI test **b** was performed 5 min after OF test. MK-801-treated animals showed decreased social activity (*F*_2, 25_ = 9.548, *P* < 0.001). *n* = 14, 6, and 8 for control, ZL006 and MK801, respectively. **c** In NORT, animals received i.p. injections immediately after the familiarization trial and the testing trial was conducted after a 3 h ITI. Discrimination index expressed by different groups of rats during the testing trial showed that MK-801 treated rats displayed deficits in the discrimination behavioral (*n* = 11, 9, and 9 for control, ZL006 and MK801, respectively; *F*_2, 26_ = 5.993, *P* < 0.01). **d** In the Y-maze test, the animals received i.p. injections immediately after the familiarization trial and the testing trial was conducted after a 1 h ITI. *n* = 6 for each group. Left: rats in the vehicle and ZL006 treated groups visited novel arm more than the other two arms (*P* < 0.05); however, animals treated with MK-801 visited novel arm less that the other arms (*P* < 0.05); Right: controls and ZL006 treated rats spent more time in the novel arm than the other arms (*P* < 0.01); no arm difference was found in the rats treated with MK-801 (*P* > 0.05). *P < 0.05, **P < 0.01; ITI inter-trial interval, OF open field, SI social interaction, NORT novel object recognition

We tested motor activity and short-term memory effects of either blocking NMDA receptors or disrupting PSD95-nNOS interactions, utilizing MK-801 (0.1 mg/kg i.p.) or ZL006 (10 mg/kg i.p.), respectively, both at doses that were equally effective in reducing fear memory. In the OF test, the total distances covered in the open-field arena after ZL006 treatments were comparable to the vehicle group, whereas the total distance was significantly higher in the MK-801 group (Fig. 2a). In the SI test, while ZL006 treated animals showed comparable interaction activity with the vehicle controls, MK-801 treated animals displayed significantly reduced interaction activity (Fig. 2b). In the NORT, DI demonstrated that ZL006 did not cause deficits in the discrimination behavior when compared with the vehicle controls, whereas rats treated with MK-801 were unable to discriminate between the familiar and the novel object (Fig. 2c). To test the spatial memory, Y-maze with a two-trial test was utilized. Here, we observed that vehicle controls and ZL006 treated rats displayed an intact spatial recognition memory: these animals had a higher frequency of visits (Vehicle: *F*_2, 15_ = 4.892, *P* < 0.05; ZL006: *F*_2, 15_ = 6.628, *P* < 0.01) and longer durations (Vehicle: *F*_2, 15_ = 10.56, *P* < 0.01; ZL006: *F*_2, 15_ = 7.965, *P* < 0.01) within the novel arm than in the other arms. However, the rats treated with MK-801 demonstrated an impaired spatial recognition memory: they visited the novel arm less than the other arms (*F*_2, 15_ = 3.896, *P* < 0.05) and spent equal time in all of the three arms (*F*_2, 15_ = 0.3418, *P* > 0.05) (Fig. 2d). Collectively, our findings demonstrate that disrupting PSD95-nNOS interaction is devoid of some of the acute effects seen with NMDA receptor antagonists, such as locomotor hyperactivity, reduced social interaction, and disrupted performances in object recognition and spatial recognition.

### Fear conditioning induces a robust increase in the amygdala PSD95/nNOS binding, which is blocked by pre-administration of ZL006

**Fig. 3:**
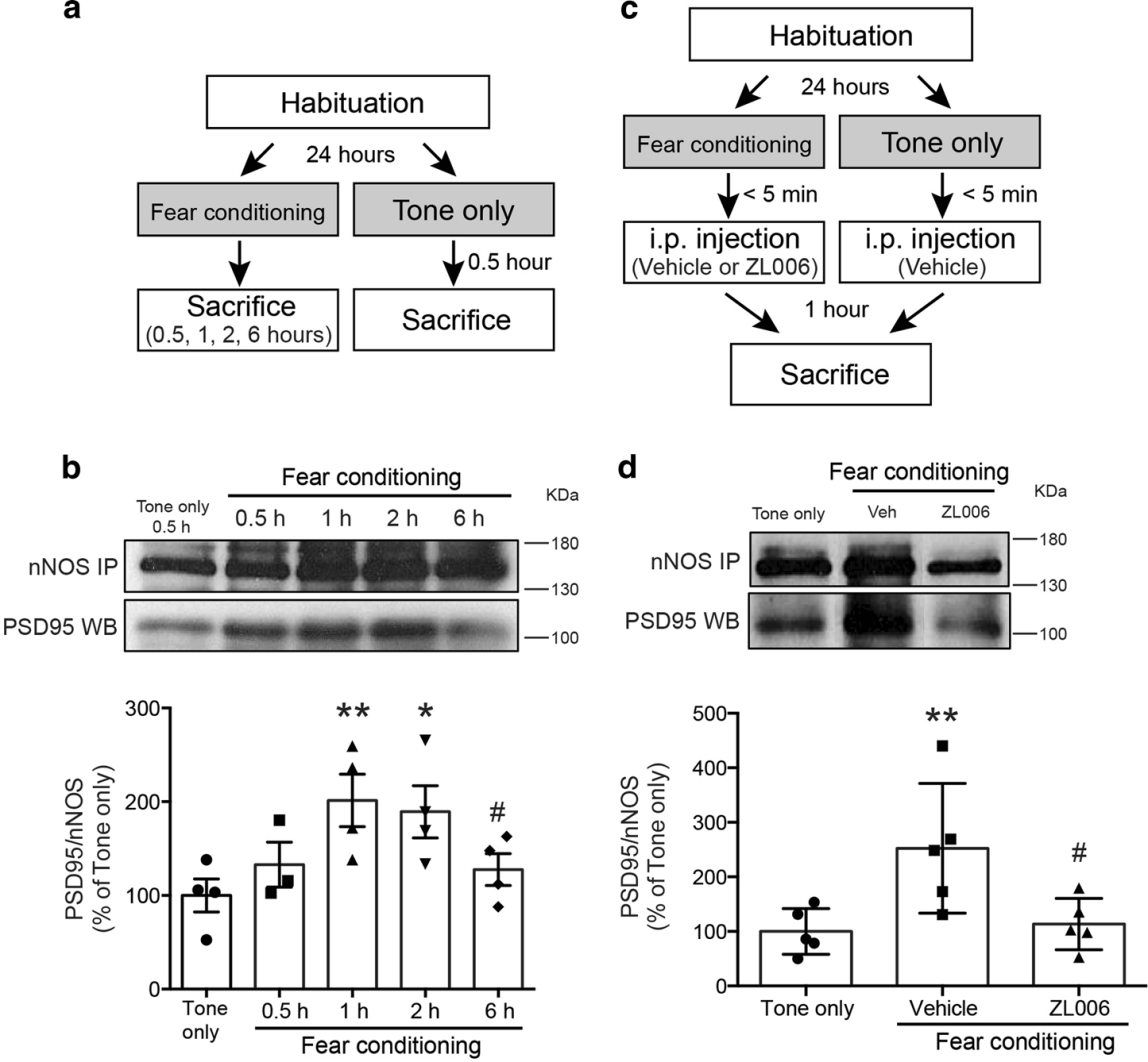
Fear conditioning induces a robust increase in amygdala PSD95/nNOS binding, which is prevented by pretreatment of ZL006. **a** Schematic of the behavioral protocol. Rats were habituated to the conditioning box, fear conditioned with 3 tone/shock pairings and sacrificed either 0.5, 1, 2, or 6 h following conditioning. The “Tone only” group did not receive shock and were sacrificed 0.5 h after non-shock training. **b** Protein extracts from BLA were immunoprecipitated with nNOS antibody and immunoblotted with PSD95 antibody (top, representative blots). Levels of PSD95/nNOS ratio were expressed as a percentage of those in “Tone only” controls (*n* = 3 or 4, *F*_4, 14_ = 3.526, *P* < 0.05) (bottom). *P < 0.05, **P < 0.01 relative to “Tone only” group; # *P* < 0.05 relative to 1 h group. **c** Schematic of the behavioral protocol. Immediately after fear conditioning, rats received i.p. injections of either vehicle or 10 mg/kg ZL006 and were sacrificed 1 h after fear conditioning. **d** Protein extracts from BLA were immunoprecipitated with nNOS antibody and immunoblotted with PSD95 antibody (top, representative blots). Levels of PSD95/nNOS ratio were expressed as a percentage of those in “Tone only” controls (*n* = 5, *F*_2, 12_ = 5.895, *P* < 0.05) (bottom). **P < 0.01 relative to “Tone only” group; # *P* < 0.05 relative to vehicle group

In the first experiment, we tested if conditioned fear training would increase PSD95-nNOS interactions in the amygdala, and determined the time course for the interaction. We observed a significantly increased association between PSD95 and nNOS at 1 h and 2 h after training, when compared with “Tone only” controls (Fig. 3a, b). The increased association between PSD95 and nNOS recovered to baseline level by 6 h. All of the fear conditioned groups showed similar acquisition of conditioned fear (Supplementary Fig. S2a). Next we tested if the fear-conditioning-induced increases in PSD95/nNOS complex could be blocked by a pretreatment of ZL006. Two groups of rats received fear conditioning and were treated with an i.p. injection of either vehicle or ZL006 10 mg/kg immediately after conditioning. The levels of PSD95/nNOS complex in the BLA were quantified with CO-IP 1 h after conditioning, the time point when the PSD95/nNOS interaction peaked after acquisition of conditioned fear as previously noted (Fig. 3c). Once again, the level of PSD95/nNOS complex was significantly increased at 1 h after conditioning and this robust increase was blocked in the rats treated with ZL006 (Fig. 3d). All three groups also showed comparable conditioned freezing response during acquisition (Supplementary Fig. S2b). Taken together, these findings suggest that fear conditioning causes a robust increase in the amygdala PSD95/nNOS binding, and that administration of ZL006 can effectively block this increased PSD95/nNOS binding.

### Disrupting PSD95/nNOS interaction prevents LTP of BLA neurons

**Fig. 4:**
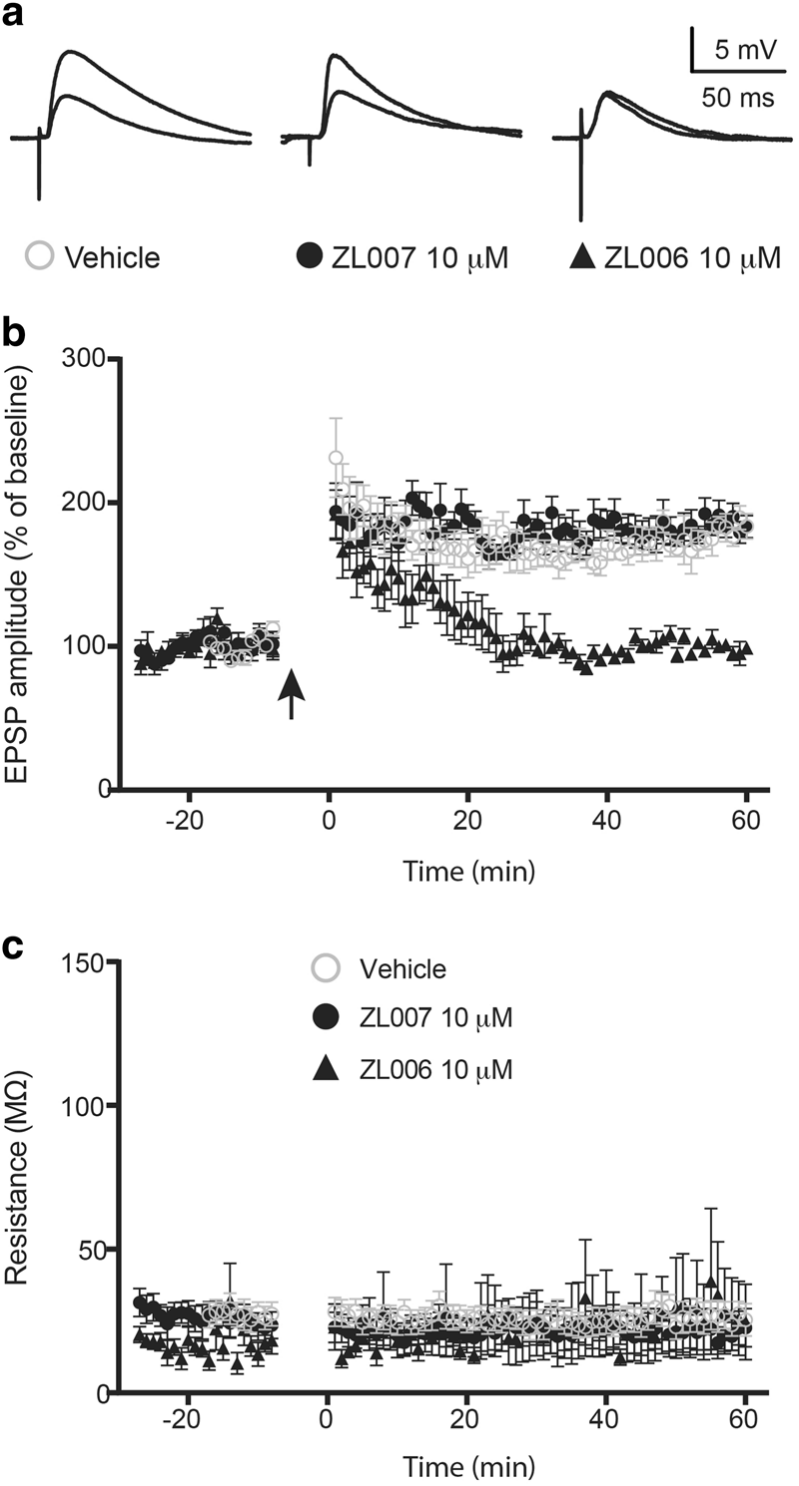
PSD95/nNOS binding inhibitor ZL006 prevents HFS-induced LTP of BLA neurons. **a** Representative traces from individual experiments before and 1 h after HFS **b** LTP produced in the ZL006 treated cells following HFS was significantly depressed compared with vehicle and ZL007 conditions (Treatment: *F*_2, 1243_ = 434.0, *P* < 0.0001). Multiple comparisons post hoc analysis revealed that EPSP responses in cells treated with ZL006 were different from cells treated with vehicle or ZL007 at all time point *t* > 16 minutes (*P* < 0.05) (*n* = 10, 6 and 6 for vehicle, ZL007 and ZL006, respectively). **c** Membrane resistance was not different when compared between groups or over time (*n* = 8, 6, and 5 for vehicle, ZL007 and ZL006, respectively). Arrow indicates initiation of high frequency stimulation (HFS)

At resting conditions, perfusion of BLA slice preparations with ZL006 had no significant effects on evoked excitatory postsynaptic potentials or input resistance during patch clamp studies of amygdala neurons in slice preparations. After establishing baseline responses to positive current injection, HFS-induced LTP was observed in vehicle and ZL007 control conditions (183.4 ± 7.8 and 185.6 ± 8.4 percent of baseline at 1 h, respectively). Following short-term potentiation, evoked EPSP responses of ZL006 treated BLA neurons gradually returned to baseline levels (98.0 ± 3.9 percent of baseline at 1 h) and were statistically divergent from control responses at all time points *t* > 16 min (Fig. 4a, b). Negative current was injected once per minute to test whether changes to evoked EPSP amplitude resulted from changes to membrane resistance and revealed no differences between conditions or over time (Fig. 4c). Collectively, these observations indicate that disrupting PSD95/nNOS binding by ZL006 impairs LTP, a cellular signature of synaptic plasticity in BLA neurons.

### Inhibiting PSD95/nNOS binding directly in the amygdala reduces conditioned fear

**Fig. 5:**
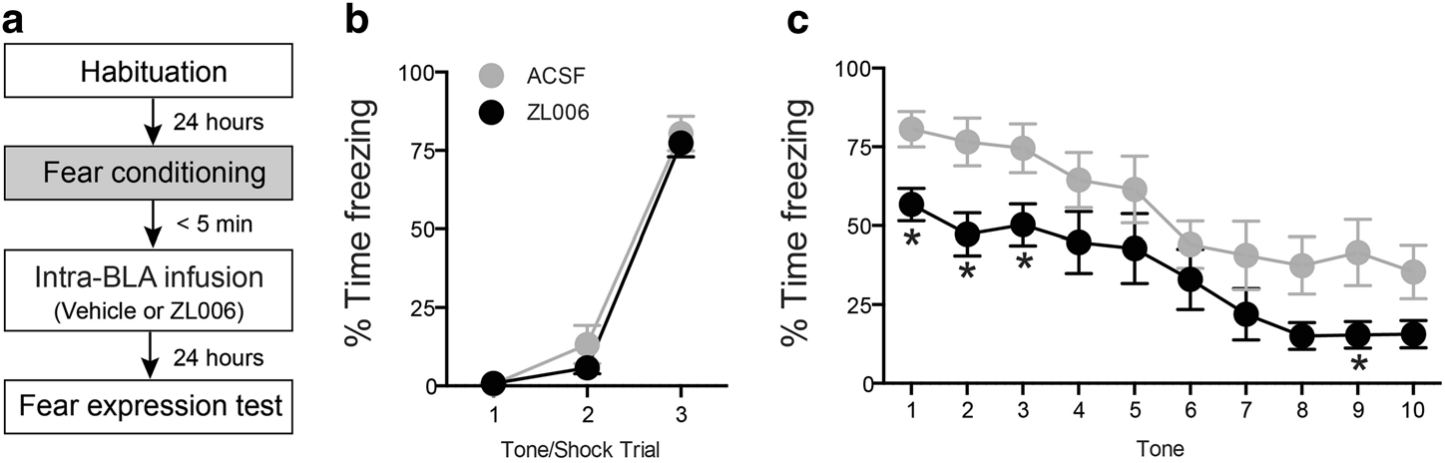
Intra-amygdala infusion of ZL006 impairs memory formation of auditory fear conditioning. **a** Schematic of the behavioral protocol. Rats were given intra-BLA infusion of ACSF (*n* = 10) or 10 µM ZL006 (*n* = 9) immediately after fear conditioning and the retention of conditioned fear memory was tested 24 h later. **b** Both pre-assigned vehicle and ZL006 groups of animals showed normal cued fear acquisition (Trial: *F*_2, 34_ = 308.8, *P* < 0.0001). **c** Animals treated with ZL006 showed significantly reduced freezing responses in the fear expression test (Treatment: *F*_1, 17_ = 4.974, *P* < 0.05). *P < 0.05 relative to vehicle

During conditioning, no difference was found in the freezing response between the pre-assigned vehicle and ZL006 group (Fig. 5b). However, in the fear expression test, animals received intra-BLA infusions of ZL006 showed significantly reduced freezing responses compared with vehicle controls (Fig. 5c). Thus, similar to systemic disruption of PSD95/nNOS binding, intra-BLA administration of small molecule inhibitor ZL006 immediately after training impairs memory formation of auditory fear conditioning. In three additional animals, the injection sites were anterior to the BLA, and ZL006 injections at these sites had no significant effects (data not shown).

## Discussion

The findings reported here clearly support that disrupting PSD95-nNOS interaction with ZL006 given shortly after an auditory cue-induced conditioning session impairs fear memory consolidation. This is consistent with a recent study that reported that ZL006 pretreatment blocks contextual fear conditioning^43^. Similar to ZL006, disruption of conditioned fear was also observed with NMDA receptor antagonist MK-801. However, unlike the NMDA antagonist, ZL006 does not appear to affect locomotor function, social interaction, and other short-term memory tests. By using co-immunoprecipitation techniques, we also demonstrate that fear conditioning results in significant increases in PSD95/nNOS binding within the amygdala in a time dependent manner, and that ZL006 pretreatment disrupts this binding. Utilizing brain slice electrophysiology, we determined that ZL006 disrupts LTP in the amygdala. Finally, disruption of PSD95/nNOS binding directly within the amygdala was able to attenuate fear. Thus, PSD95-nNOS binding appears to be a key molecular step in regulating synaptic strengthening, LTP and fear memory formation within the amygdala. Disruption of PSD95/nNOS interaction could therefore be a more targeted approach to reducing fear consolidation in the amygdala circuits during high neurotransmission state without affecting other NMDAR-dependent signaling pathways.

An important observation in our study was that ZL006, unlike MK-801, selectively disrupted fear conditioning without affecting short-term memory tests and motor responses. We found that i.p. injection of MK-801 at the dose effective in reducing fear (0.1 mg/kg) caused hyperlocomotion in rats in an OF test. Impaired locomotor function was also reported in animals treated with 7-Ni, an inhibitor of NOS^21,22^. In a SI test, we observed reduction of social behaviors with MK-801, which was in agreement with a previous study demonstrating a defective social interaction by MK-801 in a dose-dependent manner^44^. 7-Ni has been reported to possess anxiolytic effect in the SI test^45^, our experiment indicated no anxiolytic effect of ZL006.

Due to numerous studies demonstrating learning deficits caused by NMDAR antagonists and NOS inhibitors in multiple hippocampal memory tests, we investigated whether ZL006 affects hippocampus-dependent memories by utilizing NORT and Y-maze test. Consistent with previous research^46–48^, we found that post-training i.p. administration of MK-801 disrupted animals’ performance in both of the tests. However, ZL006 did not cause deficits in these tests. Overall, our results are consistent with the findings that ZL006 does not affect motor function^29,49^, spatial memory^28^, or source memory^49^ in rodents. This lack of acute motor and cognitive effects of ZL006 has significant clinical implications. NMDA antagonists, specifically ketamine, appears to be an effective treatment for reducing PTSD symptoms^50^. However, a major limitation of drugs like ketamine is their acute effects on cognition and mental status that could last several hours to days after an initial administration. A novel target that could have the same magnitude of effects in reducing conditioned fear but without such acute CNS adverse effects could address an important barrier to developing novel compounds as treatment options for fear disorders.

Our current data showed that disruption of PSD95/nNOS interaction by ZL006 selectively impairs fear memory through an amygdala-dependent mechanism without significantly influencing non-fear, acute memories as measured by NORT and Y-maze behaviors. This selectivity of ZL006 in disrupting amygdala-based fear learning tests but not primarily hippocampus-based spatial learning tasks is interesting. In agreement with our observation with ZL006, a previous study demonstrated that performance in NORT and water maze tests remained intact after administering TRIM, a selective nNOS but not an eNOS inhibitor^26^. However, these hippocampus-dependent learning mechanism became disrupted when both nNOS and eNOS were inhibited with 7-Ni, a non-selective NOS inhibitor^26^. Similarly, another electrophysiological study demonstrated that LTP in the CA1 region of the hippocampus from nNOS knockout mice and eNOS knockout mice were normal, but LTP was severely disrupted in nNOS and eNOS double mutants^51^. One possible explanation for the lack of ZL006 effects on these acute spatial or object memory tests is that in the hippocampus, eNOS activation may be compensating for the disruption of nNOS activity.

Consolidation of fear is the process where stimulus-fear association via CS/US pairing is stabilized into a persistent memory. This process is generally thought to require gene transcription and translation of post- and/or pre-synaptic proteins that result in long-term plasticity within the neural network involved in fear^3^. Previous studies have suggested that the NO signaling pathway plays a critical role in facilitating this long-term plasticity. For example, biochemical and behavioral studies have shown that intra-amygdala infusion of the NOS inhibitor 7-Ni or PKG (a downstream effector of NO) inhibitor Rp-8-Br-PET-cGMPS significantly reduced the fear-conditioning-induced expression of postsynaptic GluR1, pre-synaptic synaptophysin, and synapsin in the amygdala^16^; animals receiving infusions of the above drugs also exhibited impaired conditioned fear memory^15,17^. Previous studies have also revealed physical association of nNOS with a variety of regulatory proteins and these associations are critical in regulating nNOS activity and the resulting NO production. PSD95 is one of the regulatory proteins that interact directly with the PDZ domain of nNOS^52^. In the present study, we first investigated if PSD95/nNOS interaction is induced by fear conditioning utilizing a CO-IP assay. We found that amygdalar PSD95/nNOS interaction began to increase by 30 min after fear conditioning, peaked at 1 h, and remained increased until 6 h after conditioning. Importantly, i.p. administration of ZL006 immediately following fear conditioning prevented the enhancement of PSD95/nNOS interaction measured 1 h after conditioning. The CO-IP results collectively suggest an involvement of increased PSD95/nNOS interaction during the consolidation phase of fear memory, and that disruption of PSD95/nNOS interaction by ZL006 blocks this process. Based on our findings with CO-IP assay and LTP study, we finally tested if direct disruption of PSD95/nNOS interaction in the amygdala could impair the consolidation of fear memory. We found that intra-BLA infusion of ZL006 at an effective dose for reducing LTP also impaired fear memory expression when administered immediately after conditioning.

These results are consistent with the idea that the amygdala may be the neural locus mediating ZL006 action on auditory fear conditioned responses. However, the degree to which intra-BLA ZL006 injections were able disrupt the consolidation of fear was much lower than that observed when we administered the same drug systemically. This suggests the possibility that in addition to amygdala, other brain regions might also be involved in mediating the effects of ZL006 on conditioned fear. A recent study reported that ZL006 acting through the hippocampus could disrupt contextual fear memories^43^. Thus, hippocampus appears to be another key region where PSD95-nNOS interaction is critical for certain subtypes of fear responses. The hippocampus is also implicated in other aspects of conditioned fear responses, including fear generalization and fear extinction. Similarly, regions of the prefrontal cortex are also important in the consolidation and extinction of conditioned fear^53^ and may include interactions between BDNF and NO^54^. Thus, it would be important to study the role of PSD95-nNOS interactions within these critical structures in regulating various aspects of conditioned fear including fear acquisition, extinction learning and extinction memory.

In addition to conditioned fear, this mechanism is also implicated in other neuropsychiatric and neurological disorders such as depression^55,56^, stroke^28^, Parkinson’s disease^57^, and chronic pain^29^. In a previous study, utilizing depression models, Doucet et al. observed that the antidepressant effects of ZL006 was evident only after 24 and 72 h following treatment^55^. This delayed effect of the drug on behavioral measures is also consistent with its mechanism of action based on modifying neural plasticity. Synaptic plasticity is a critical mechanism in both conditioned fear memory and extinction. This series of studies primarily focused on fear conditioning, but more future studies are needed to elucidate about the role of PSD95-nNOS interaction in extinction learning and extinction memory retrieval. In addition, we need to better understand if ZL006 effects confined to specific type of associative learning and plasticity or if it broadly pro-cognitive. If ZL006 inhibits plasticity too broadly, it could impair extinction memory given its inhibitory effects on plasticity. From our preliminary data in auditory fear conditioning, there appears to be little increase PSD95-nNOS interactions in the prefrontal cortex within 1–2 h after fear conditioning, and chronic treatment with ZL006 does not appear to disrupt learning in spatial memory or source memory tests^49^. Thus, we predict that it would most efficacious in weakening fear associations and unlikely to interfere with safety learning. Future studies will need to test these concepts in detail.

In summary, our present work shows that disrupting PSD95/nNOS interaction with the small molecule ZL006 prevents the fear-conditioning-induced increase in PSD95/nNOS interaction within the amygdala, impairs LTP in amygdala neurons and attenuates the consolidation of fear memory. Importantly, unlike NMDAR antagonists, systemic ZL006 is devoid of effects on locomotor activity, and acute effects on learning and memory, indicating that disrupting PSD95/nNOS interaction represents a novel therapeutic approach for reducing learned fear without eliciting adverse effects. Future studies will investigate the molecular and cellular mechanisms underlying ZL006 action and support the development of PSD95/nNOS interaction based treatment approach for fear-related disorders such as PTSD.

## Electronic supplementary material

Supplemental Figure S1

Supplemental Figure S2

Supplemental Figure S3

Supplemental legends
