# Supplementary figures and images for: PSD95 and nNOS interaction as a novel molecular target to modulate conditioned fear: relevance to PTSD
Source: Transl Psychiatry. 2018 Aug 14;8:155. doi: 10.1038/s41398-018-0208-5 (PMC6092346; doi:10.1038/s41398-018-0208-5)

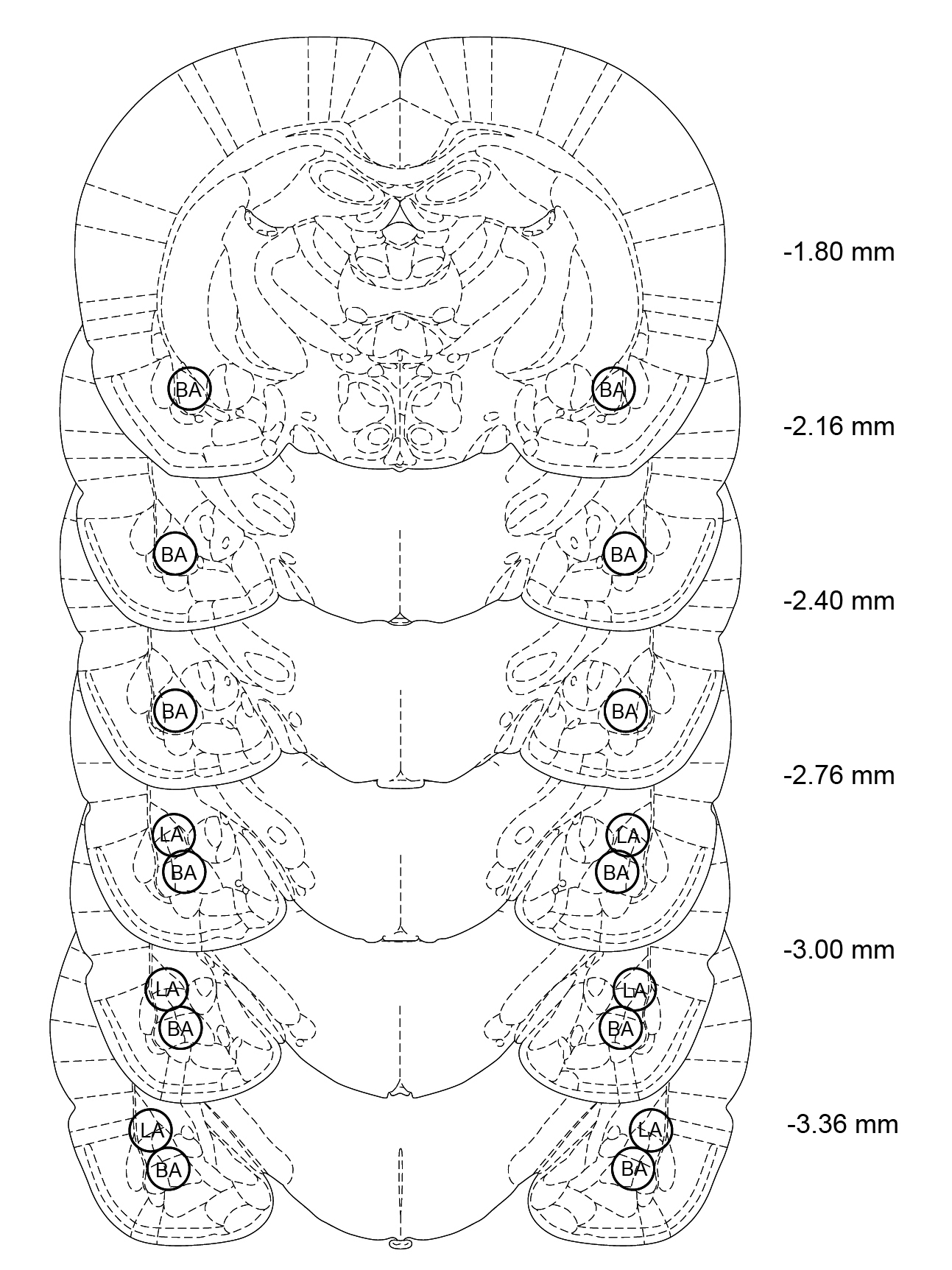

Supplement: Supplementary file 1 — Supplemental Figure S1 [file 41398_2018_208_MOESM1_ESM.jpg]

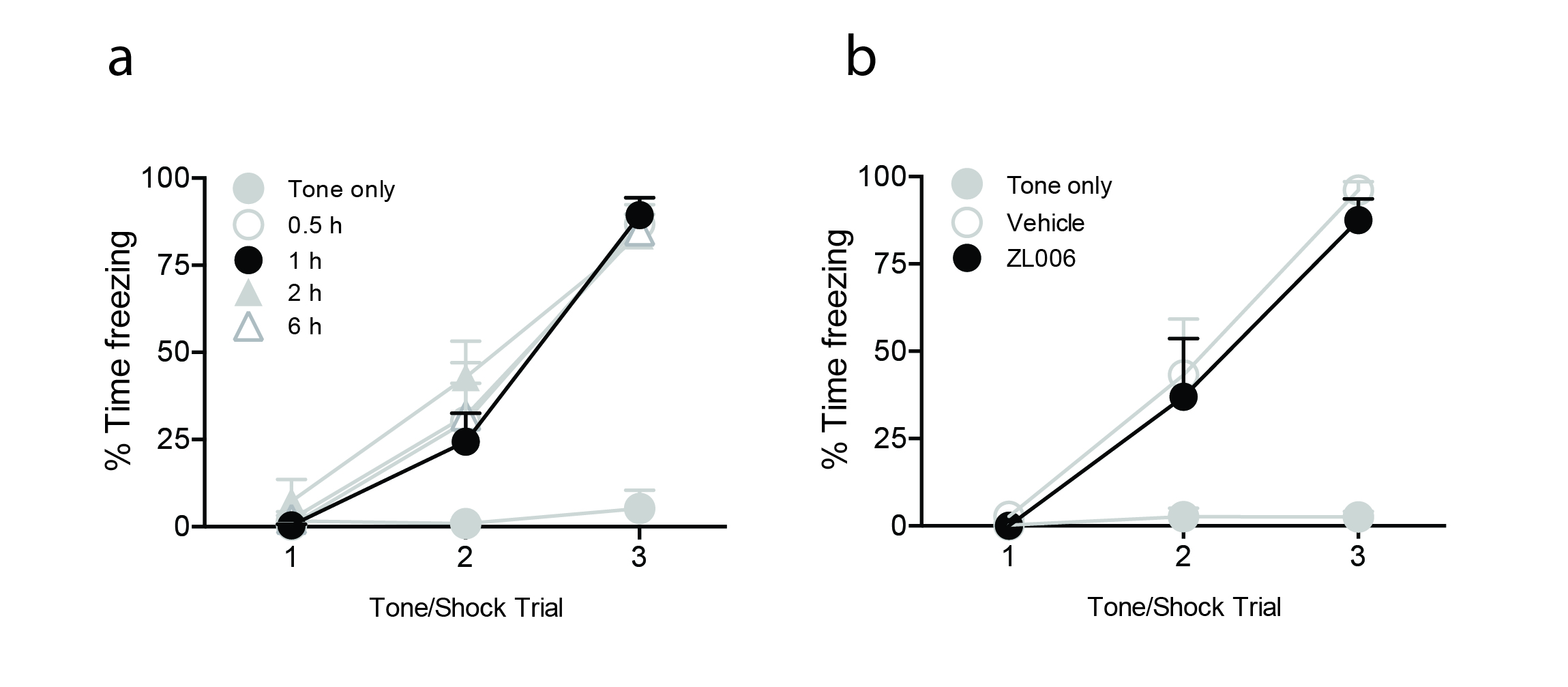

Supplement: Supplementary file 2 — Supplemental Figure S2 [file 41398_2018_208_MOESM2_ESM.jpg]

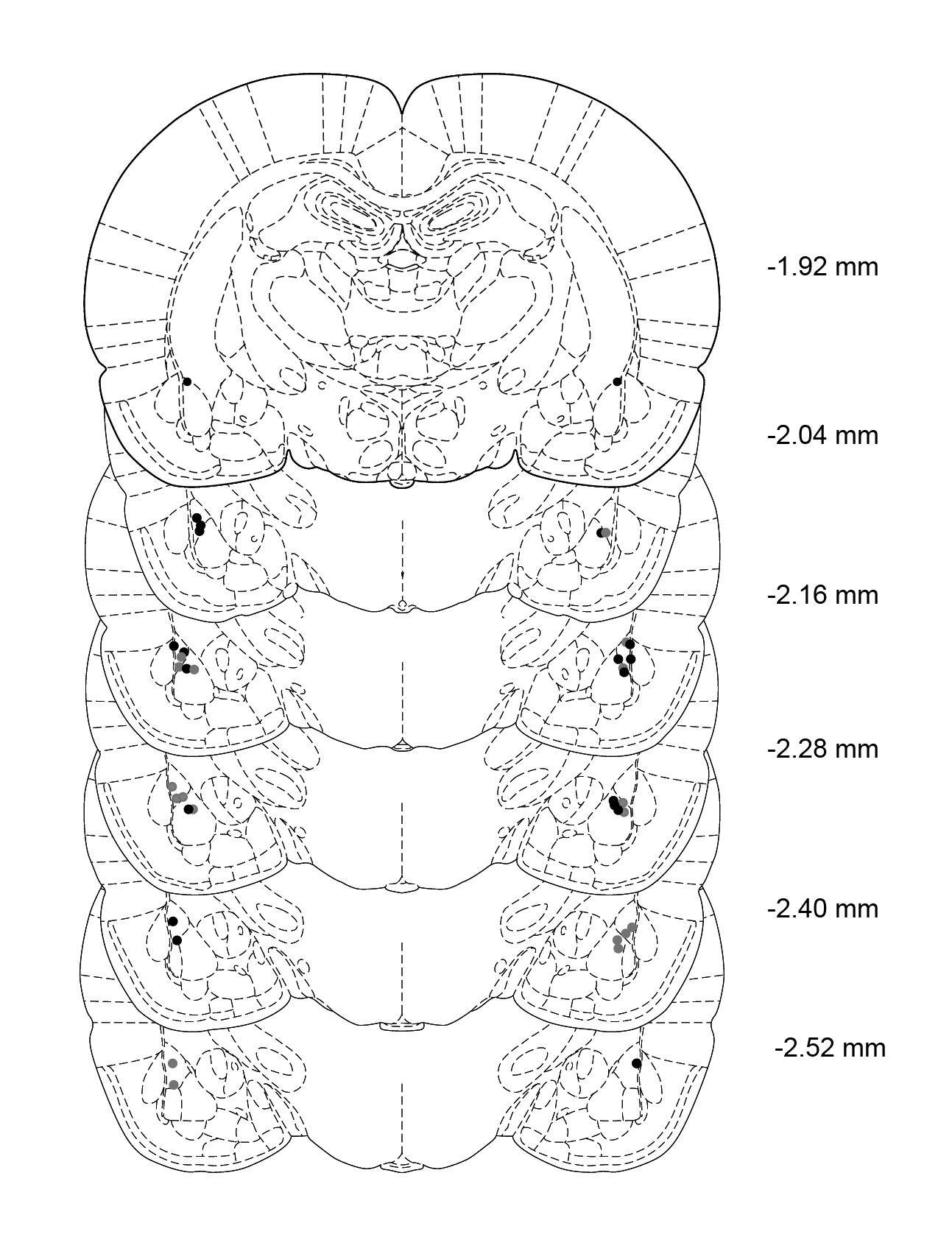

Supplement: Supplementary file 3 — Supplemental Figure S3 [file 41398_2018_208_MOESM3_ESM.jpg]
