# Supplementary material for: PSD95 and nNOS interaction as a novel molecular target to modulate conditioned fear: relevance to PTSD
Source: Transl Psychiatry. 2018 Aug 14;8:155. doi: 10.1038/s41398-018-0208-5 (PMC6092346; doi:10.1038/s41398-018-0208-5)
Supplement: Supplementary file 4 — Supplemental legends [file 41398_2018_208_MOESM4_ESM.docx]

Supplementary Figure S1. Schematic image depicting the location of the BLA punches in the CO-IP experiments. The black circles delimit the regions of the tissue punches. LA: lateral nucleus of amygdala; BA: basal nucleus of amygdala. Drawings are adapted from an atlas (Paxinos and Watson 2005).

Supplementary Figure S2. Acquisition of conditioned fear in animals used for CO-IP experiments. (A) Four groups of animals sacrificed at different time points all acquired fear normally and equivalently (Trial: F2, 22 = 237.6, P < 0.0001; Treatment: F3, 11 =0.2241, P > 0.05). Animals in ‘Tone only’ group showed no freezing responses to the tone. (B) Animals in the vehicle and ZL006 group acquired fear normally and no difference was found in the freezing level across trials between groups (Trial: F2, 16 = 55.77, P < 0.0001; Treatment: F1, 8 =0.3638, P > 0.05). Animals in ‘Tone only’ group showed no freezing responses to the tone.

Supplementary Figure S3. Histological verification of cannula placements. The symbols represent the injection sites in vehicle (black circle) and ZL006 (grey circle) treated animals that were included in the microinfusion experiment. Sections are based on the atlas of Paxinos and Watson (2005). Numbers indicate the distance from bregma (in mm).
